# A Multichannel Microfluidic Sensing Cartridge for Bioanalytical Applications of Monolithic Quartz Crystal Microbalance

**DOI:** 10.3390/bios10120189

**Published:** 2020-11-24

**Authors:** María Calero, Román Fernández, Pablo García, José Vicente García, María García, Esther Gamero-Sandemetrio, Ilya Reviakine, Antonio Arnau, Yolanda Jiménez

**Affiliations:** 1 Centro de Investigación e Innovación en Bioingeniería, Universitat Politècnica de València, 46022 Valencia, Spain; macaal3@teleco.upv.es (M.C.); rfernandez@awsensors.com (R.F.); aarnau@eln.upv.es (A.A.); 2 Advanced Wave Sensors S.L. Paterna, 46988 Valencia, Spain; pgarcia@awsensors.com (P.G.); jvgarcia@awsensors.com (J.V.G.); mgarcia@awsensors.com (M.G.); egamero@florida-uni.es (E.G.-S.); 3 Unidad de Educación, Florida Universitaria, 46470 Valencia, Spain; 4 IMBB-FORTH and Department of Biology, University of Crete, Heraklion, 70013 Crete, Greece; 5 Department of Bioengineering, University of Washington, Seattle, WA 98150, USA

**Keywords:** HFF-QCM (high fundamental frequency quartz crystal microbalance), mass transport, flow cell, biosensor, food safety, PoC (point of care), MQCM (monolithic quartz crystal microbalance)

## Abstract

Integrating acoustic wave sensors into lab-on-a-chip (LoC) devices is a well-known challenge. We address this challenge by designing a microfluidic device housing a monolithic array of 24 high-fundamental frequency quartz crystal microbalance with dissipation (HFF-QCMD) sensors. The device features six 6-µL channels of four sensors each for low-volume parallel measurements, a sealing mechanism that provides appropriate pressure control while assuring liquid confinement and maintaining good stability, and provides a mechanical, electrical, and thermal interface with the characterization electronics. We validate the device by measuring the response of the HFF-QCMD sensors to the air-to-liquid transition, for which the robust Kanazawa–Gordon–Mason theory exists, and then by studying the adsorption of model bioanalytes (neutravidin and biotinylated albumin). With these experiments, we show how the effects of the protein–surface interactions propagate within adsorbed protein multilayers, offering essentially new insight into the design of affinity-based bioanalytical sensors.

## 1. Introduction

Inexpensive, fast, parallel, small-volume sensors for detecting minute amounts of analytes in complex samples are required for wide-spread application of lab-on-a-chip (LoC) devices in research-, industrial-, and health-related areas [1,2]. Equipped with such sensors, LoC devices can overcome the drawbacks of the conventional bulk analytical approaches that rely on expensive centralized facilities and play their singular role in the individualization of health care promised by personalized medicine [3].

Quartz crystal microbalance with dissipation, or QCMD, offers the technology needed for developing and mass-producing sensors for LoC applications. QCMD is known for its thin film monitoring [4] and biomolecular interaction measurement capabilities [5]. Digital, label-free, and very simple—its basic principle is based on electrically measuring resonance properties (frequency and dissipation) of a quartz crystal resonator [6]—QCMD can be easily automated. Key challenges for integrating QCMD technology into LoC-based applications are low throughput, high cost per sensor and per assay (related to sample/reagents volume), and lack of versatile and effective microfluidic/mechanical interfaces that can assure reliable field operation. Further improvements in sensitivity would also be beneficial for rapidly detecting small amounts of analytes in dilute samples.

**Figure 1:**
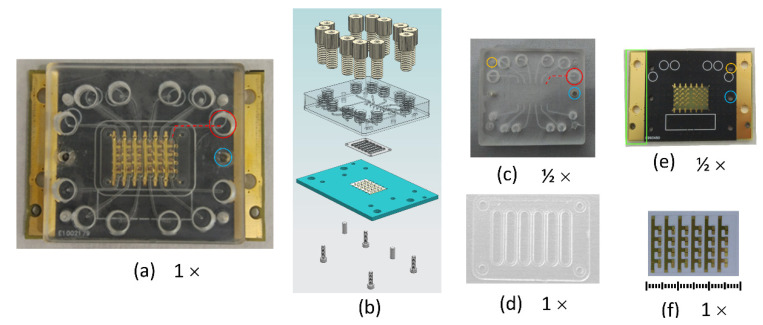
Multichannel Microfluidic Sensor Cartridge. Assembled cartridge (**a**). Schematic of cartridge assembly (**b**) from components (from top to bottom): microfluidic connectors, Poly(methyl methacrylate) (PMMA) cell, Poly(dimethylsiloxane) (PDMS) gasket, Printed Circuit Board (PCB) with the array, assembly screws and alignment pins. Photos of its components: PMMA cell (**c**), PDMS gasket with six channels (**d**), PCB (**e**), and the 24-element HFF-QCMD array (**f**, see Figure S1 in the Supporting Information for an enlarged view). Note the difference in scale: (**c**,**e**) are shown at half the size relative to (**a**,**d**,**f**), which are drawn to scale. Array dimensions are 14.25 mm × 9.05 mm.

To address these challenges, we introduce a microfluidic cartridge housing a monolithic high fundamental frequency (HFF) QCMD sensor array. We picked HFF-QCMD sensors because they are more sensitive than their low fundamental frequency counterparts, and can be used with low sample volumes (a few μL), and because they offer the possibility of integration into monolithic arrays [7,8,9]. Higher sensitivity is due to the smaller thickness of the HFF resonators [4,10], which in turn leads to a reduced surface area and therefore, to smaller sample volume [11]. Integration overcomes two major impediments for developing QCMD-based sensors for LoC applications: low throughput and relatively high individual sensor cost. QCMD and HFF-QCMD sensors have already been used to develop immunoassays [12,13,14,15,16,17] and DNA hybridization assays with the limit of detection (LoD) in the tens of femtomoles [18] and in complex samples (blood, saliva) [19,20], but currently, advanced commercial systems offer measurements with at most four sensors at a time, each of which has to be operated individually. This negates other advantages of QCMD technology in high-volume LoC applications. To this end, we have recently reported our design of monolithic 150 MHz HFF-QCMD sensor arrays and characterized their performance [7]. Here, we present the design of an integrated microfluidic cartridge for housing the arrays (Figure 1) and evaluate its performance for measuring biomolecular interactions in a biotin/neutravidin based system. Our work advances the state-of-the-art by integrating sensing and microfluidic analyte delivery compatible with LoC applications.

The development of robust and simple HFF-QCMD detection systems for LoC applications is very much in its infancy [2]. Integration of the fluidic circuitry with the array of quartz sensors faces a number of challenges [21,22], the key of which is insuring independent operation of the individual sensor elements that is free of the interaction between them. Interactions between sensors can be mediated electrically, mechanically, or via the fluidic path affecting analyte transport to the surface. Therefore, our evaluation of the cartridge and array performance focused as much on the reproducible measurements of frequency and dissipation shifts in response to the introduction of bioanalytes as on the independent operation of the individual array elements, as evidenced by the absence of artifacts originating from the interaction between them through any of the three pathways (electrical, mechanical, fluidic). Our experimental results were consistent with the literature and with the results of numerical calculations of molecular transport rates based on the channel and array geometry, molecular diffusion coefficients, and fluid flow rates.

## 2. Materials and Methods

### 2.1. Materials

Nanopure water used in this study was either analytical grade water (Panreac Química SLU, Barcelona, Spain), or produced with a Smart2Pure UVUF water purification system (Thermo Fisher Scientific, Barcelona, Spain). Nitrogen was from Air Liquide España S.A. (Valencia, Spain). Phosphate buffered saline (PBS) tablets for preparing 0.01 M phosphate buffer containing 0.0027 M potassium chloride and 0.137 M sodium chloride, pH 7.4, at 25 °C, and Bovine Serum Albumin (BSA) were purchased from Sigma Aldrich Química, S.L.U. (Madrid, Spain). NeutrAvidin (Nav), biotinylated BSA (bBSA), and Sodium Dodecyl Sulfate (SDS) 20% solution were purchased from Fisher Scientific S.L. (Madrid, Spain). COBAS Cleaner was purchased from Sanilabo S.L. (Valencia, Spain).

Poly(methyl methacrylate), PMMA, was from Monje Hermanos S.L. (Valencia, Spain); poly(dimethylsiloxane), PDMS, was from Ellsworth Adhesives Iberica, (Madrid, Spain). Single-component conductive epoxy AA-DUCT 900 was from Atom Adhesives (Providence, RI, USA). DuPont™ Teflon^®^ FEP film with a thickness of 76 µm was from Dupont (Wilmington, DE, USA).

### 2.2. Multichannel Microfluidic Sensing Cartridge Design

The multichannel sensor cartridge consists of an array, a custom-made Printed Circuit Board (PCB) for mounting the array, and a microfluidic cell consisting of a gasket and the body. These components are shown in Figure 1 while their design and manufacturing are described below.

### 2.3. Arrays and PCBs

Monolithic HFF-QCMD sensor arrays (AWS-Array2-24-150.0M, Advanced Wave Sensors (AWSensors) S. L., Valencia, Spain) were manufactured as described previously [7]. They were mounted on rectangular 52.02 mm × 36.02 mm × 1.55 mm PCBs custom-designed using the ALTIUM Designer 18 software package (Altium, San Diego, CA, USA) following high-frequency signal routing and crosstalk prevention considerations and manufactured from FR4-type material by Eurocircuits (Mechelen, Belgium). The mounting was done with a single-component conductive epoxy that was deposited by stencil printing technology (eC-stencil-mate, Eurocircuits). The stencil was configured to leave a tiny quantity (≈0.16 mg) of epoxy on each contact area, sufficient for making electrical contact, but not in excess in order not to affect the resonant behavior of the sensors. Before the deposition of the epoxy, the PCBs were degreased with acetone and dried at 50 °C for 5 min. Once the conducting epoxy was deposited, the arrays were placed on the PCBs manually with the assistance of a USB Microscope (Shenzhen Andonstar Tech Co. Ltd., Shenzhen, China) and a manual pick and place machine (eC-placer, Eurocircuits). The epoxy was cured at 150 °C for 1 h in an oven (eC-reflow-mate v4, Eurocircuits, Mechelen, Belgium). During the curing process, a 70 g weight was placed over the array to ensure a good contact between the array and the PCB. The array surface was protected with the transparent Fluorinated Ethylene Propylene (FEP) film.

### 2.4. Microfluidic Cell

The microfluidic cell consisted of two parts: a flexible gasket containing six independent flow channels, one for each column of the array, and the body of the cell for housing the gasket that contained inlet and outlet fluidic channels, the opening for the mounting screws, and the alignment pins (Figure 1b).

The gaskets were fabricated from PDMS using a mold, 9 gaskets at a time. To this end, the required volume of the PDMS curing agent and PDMS monomer was mixed at the 1:10 ratio. Vacuum was applied to the mixture for 45 min to remove air. The mold was dried at 60 °C and the freshly degassed PDMS mixture was cast onto the mold. Curing took place in an oven at 60 °C for 80 min. The height of the channels is determined by the thickness of the gasket being 480 µm while the width is 1.46 mm. Consequently, the total volume of each fluidic channel was less than 6 μL. (See Section S3 of the Supporting Information).

The body of the microfluidic cell consisted of two parts: the top that contained the openings for standard microfluidic connectors (VacuTight Ferrule Tefzel Red P-840 (ETFE) 1/16, Fitting P-844, IDEX Health & Science, Rohnert Park, CA, USA) for connecting the cell to the external flow control device, and the bottom, that contained inlet and outlet microchannels, 12 in total, connecting those openings to the fluidic channels in the gasket.

The mold for manufacturing PDMS gaskets, and the two parts of the microfluidic cell, were manufactured from PMMA in the vertical CNC (Computer Numerical Control) machining center (Chevalier 1418VMC-Plus, Falcon Machine Tools Co. Ltd., Chang Hua, Taiwan). The two pieces of the microfluidic cell were joined by first softening their surfaces with ethanol, and then pressing them against each other over a period of 1 min at 70 °C, as described in ref. [23]. In this manner, a one-piece microfluidic cell was obtained. The PDMS gasket was mounted on the cell, and the cell with the gasket was placed on top of the PCB-mounted array. The assembly was guided by the alignment pins and held together with screws, as shown in Figure 1b.

### 2.5. Evaluation of Cartridge Performance

The performance of the array-based sensor cartridge was evaluated by monitoring resonance frequency and dissipation in situ and in real time as the arrays were exposed to liquid flow and as proteins were adsorbed to the surfaces of the arrays (in separate experiments). The results of the protein adsorption experiments were further compared with those obtained with the commercially available, individual 50 MHz HFF-QCMD sensors (AWsensors S.L., Valencia, Spain).

Resonance frequency and bandwidth (or dissipation) of the arrays and individual HFF sensors were measured as a function of time with the AWSensors X24 and A20+ platforms, respectively, running the AWSuite software package. Individual sensors were mounted in the standard, commercially available QuickLock^®^ measurement cells (AWSensors S.L., Valencia, Spain). Fluid flow and sample injection were controlled with the AWSensors multichannel F20+ fluidics controller connected to the inlets of the sensing cartridge (for the arrays) or the inlets of the QuickLock^®^ measurement cells.

For the measurements of the air-to-liquid transition, baseline frequency and bandwidth signals were acquired in air for ~15 min, followed by flowing PBS buffer, at a flow rate of 20 μL/min.

For the protein adsorption experiments, baseline frequency and dissipation signals were acquired in buffer flowing at a rate of 14.5–20 μL/min, depending on the experiment, for ~5–10 min, followed by the injection of the proteins, Nav (at a concentration of 100–200 μg/mL in PBS) or bBSA (at a concentration of 100 μg/mL). Each injection was always repeated twice, 100 μL each time, to ensure that adsorption reached saturation.

### 2.6. Array, Cell and Sensor Cleaning and Preparation

Prior to the experiments, PCB-mounted arrays and individual sensors were immersed in freshly filtered 2% SDS solution for 30 min. They were rinsed with Nanopure water and dried under a stream of nitrogen directed at the array and sensor surfaces through a 0.45 μm pore diameter filter housed in hand-held filter gun assembly (Skan AG, Allschwil, Switzerland) and then cleaned for 30 min in a UV/Ozone Cleaner (BioForce Nanosciences, Salt Lake City, UT, USA) that was preheated for 30 min prior to use. After cleaning, array and sensor surfaces were rinsed with ethanol to reduce the oxide that forms as a result of the UV-Ozone treatment [24] and once again dried with a stream of filtered nitrogen.

The PDMS gasket and the PMMA cell were immersed in COBAS cleaner for 30 min, rinsed with, and then sonicated in Nanopure water for 15 min to remove traces of the detergent, and dried with a stream of filtered nitrogen.

Individual sensors were mounted in the QuickLock™ measurement cells cleaned with the COBAS cleaning solution for 30 min, followed by repeated rinsing with water and ethanol, and drying with a stream of filtered nitrogen.

## 3. Results and Discussion

### 3.1. Multichannel Microfluidic Sensing Cartridge Design and Assembly

A microfluidic cartridge holding a 24 HFF-QCM sensor array operating at 150 MHz [7] was designed and implemented (Figure 1a). It consists of a microfluidic cell that delivers fluid to the sensing elements arranged in six channels of four elements each (Figure 1c), a soft PDMS gasket forming the six microfluidic channels (Figure 1d) that seals the PMMA fluid cell to the array, and the array mounted on the PCB that served as the bottom of the cell (Figure 1e,f). PMMA is a low-cost, transparent material with good thermal insulating capacity that is suitable for disposable cartridges and is used routinely in biomedical applications (e.g., bone cement [25,26]). It has been selected because of its adequate rigidity to confer robustness to the microfluidic assembly design and its mass fabrication potential [23]. The PDMS gasket is fixed in the PMMA cell with the help of four guides (Figure 1d). The PMMA cell contains sockets for the fluidic connectors on the top (outlined in red in Figure 1a,c), as well as sockets for the guiding pins (blue in Figure 1a,c,e) used to align the array, and mounting screws (yellow in Figure 1c,e) used to fix it. The assembly process is very simple and results in a rectangular chamber design which avoids non-uniform sample distribution [27,28]. The PCB offers mechanical protection, ease of handling, improves the thermal stability of the array during measurements, and forms the contacts between the array and the acquisition electronics. To this end, we integrated into the PCB design two gold sidebands (highlighted in a green rectangle in Figure 1e) that are connected to the Peltier elements of the temperature control system, providing a direct thermal path. Our design allows simultaneous measurements with six different solutions. Furthermore, within each of these six measurements, it is possible to have four replicas with identically treated array element surfaces. Alternatively, each array element could be used to immobilize a different biomolecule, allowing four independent measurements with each of the six solutions at the expense of statistics.

### 3.2. Cartridge Performance

The performance of the cartridge was evaluated in two separated sets of experiments: the responses of the sensors to changing the media from air to liquid were measured in a first set of experiments, and sequential adsorption of proteins neutravidin and biotinylated BSA (bBSA) on the sensor surface was followed in the second set. Cartridge and array performance were evaluated by comparing our experimental results with theoretical predictions and with other experimental data, either obtained in this study with individual HFF-QCMD sensors, or reported in the literature with the classical low-fundamental frequency QCMD sensors. The distribution of sensor responses was analyzed as a function of sensor position in the array to assure independent operation of the individual sensors. Finally, biomolecular transport in the cartridge has also been studied to evaluate possible limitations of the microfluidic cartridge design.

#### 3.2.1. Air-to-Liquid Transition

**Figure 2:**
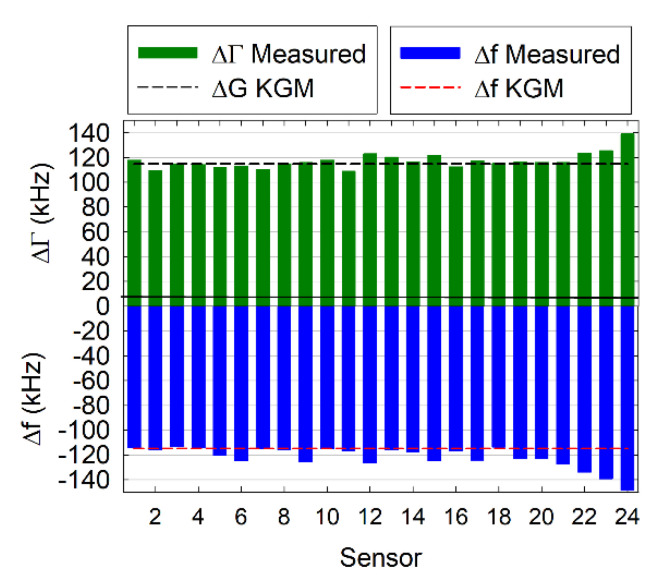
Air-buffer measurements. A plot of the typical set of air-to-buffer shifts for all the elements of one array (bars) with the expected values calculated based on the Kanazawa-Gordon-Mason equation (dashed lines).

Changes in the frequency and bandwidth of a quartz resonator upon exposure to a Newtonian liquid are well-understood as a result of the classical works by Mason [29] and Kanazawa [30]. When discussing the air-liquid shifts, it is more convenient to use bandwidth than dissipation, because the frequency and bandwidth changes are expected to be equal and opposite: $∆f=-∆\Gamma =-f_{res}^{3/2}{\left(\eta_{l}\rho_{l}/\pi \rho_{q}\mu_{q}\right)}^{1/2}$; this is the so-called Kanazawa–Gordon–Mason equation, where $f_{res}$ is the fundamental resonance frequency of the resonator, $\eta_{l}$ and $\rho_{l}$ are the liquid viscosity and density, respectively, $\rho_{q}$ is the density of quartz, and $\mu_{q}$ is its shear elastic modulus. Experimental results are shown in Figure 2, where the shifts in both signals upon the transition from air to buffer are plotted as a function of the array element (Supplementary Figure S1). We find no systematic trends in the shifts of either of the two signals as a function of the array element position, as is expected for the normal functioning of the array, the flow system, and the electronics. It is interesting to note the increase in ∆f and −∆Γ in sensors 20 to 24. Although it seems a systematic trend, this effect was not observed in other measurements, confirming its coincidental nature.

On average, we observed shifts of −120 ± 15 kHz for ∆f and 115 ± 9 kHz for ∆Γ, averaged over 110 individual air-to-buffer measurements. The theoretical values for the arrays used in these measurements are ± (115 ± 2) kHz, where the negative and the positive signs refer to the frequency and the dissipation shifts, respectively, and the error arises from the variation of the elements’ resonance frequencies [7]. The larger experimentally observed value of ∆f is expected, because finite roughness typically masquerades as an additional Sauerbrey shift that does not affect the bandwidth [31,32]; a 5 kHz difference in frequency would correspond to a Sauerbrey film of ~9.9 Å with a density of 1 g/cm^3^, which is reasonable given the roughness values of ~1 nm we reported previously for the surfaces of these arrays [7].

#### 3.2.2. Protein Adsorption and Interaction Studies

The functionality of the multichannel cartridges for sensing biomolecular interactions was evaluated using neutravidin (Nav) and biotinylated BSA (bBSA) as a model system that is widely used in biotechnology and bioanalytics [33,34].

**Figure 3:**
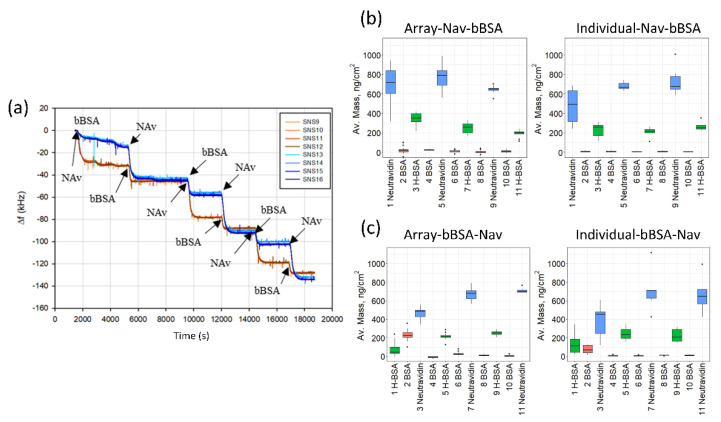
Protein adsorption measurements. (**a**) Frequency changes due to the sequential adsorption of proteins for two columns of the array relative to a baseline in buffer. In one of the two columns (SNS13 to SNS16), proteins were adsorbed in sequence bBSA-Nav- bBSA-Nav- bBSA-Nav, in the other (SNS9 to SNS12) Nav-bBSA-Nav-bBSA- Nav-bBSA. SNSi with *i* = 9 to 16 refers to the element position on the array according to Figure S1 in the Supporting Information. (**b**,**c**) Mass changes observed in the experiments performed with arrays (**left**) and individual 50 MHz resonators at the 3rd overtone (**right**) for the two adsorption sequences, respectively. Error bars are standard deviations.

Proteins were allowed to adsorb in sequence, starting with Nav in one set of experiments, and starting with bBSA in another set. Each sequence consisted of three sets of (Nav + bBSA) pairs, with non-biotinylated BSA injected between each step to test for non-specific adsorption. The purpose of the two different sequences was to establish a protocol for subsequent applications of these arrays in bioanalytical sensing. One such sequence is shown in Figure 3a.

**Figure 4:**
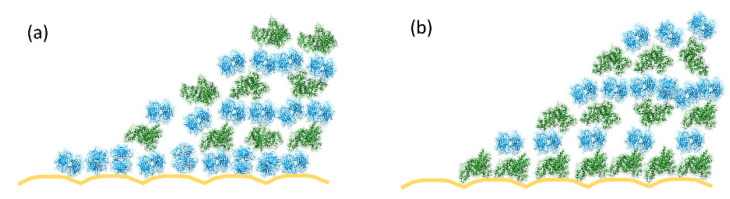
Schematic representation of the surface architecture achieved in the adsorption experiments. (**a**) Shows the adsorption sequence starting with Nav, and (**b**)—starting with bBSA. The gold is shown in yellow, Nav in light blue, and BSA in green. No distinction is made between the biotinylated and non-biotinylated BSA. The vertical dimension of the roughness of the gold is to scale with the sizes of the proteins; similarly, the protein dimensions are also drawn to scale relative to each other, but Nav aggregation is ignored for simplicity. On average, there is ~ one bBSA molecule for every two Nav molecules adsorbed on gold. Away from the surface, there are ~ three Nav molecules per every bBSA molecule. See Section S2 in the Supporting Information for further discussion. This figure was prepared using USCF Chimera version 1.14 [35].

Average results from multiple arrays and individual HFF sensors are shown in Figure 3b (for the Nav–BSA sequence) and in Figure 3c (for the bBSA–Nav) sequence. The adsorbed layer masses, calculated from the frequency shifts using the Sauerbrey relationship [4], are summarized in Table S1 in the Supporting Information. The masses expected on the basis of the sizes of the molecules and the corresponding literature values are presented in Supplementary Table S2. The adsorption processes are depicted schematically in Figure 4.

Several trends can be noted in the experimental data. First and foremost, there is a good agreement between the results obtained with the arrays and with the individual HFF resonators (Figure 3, Supplementary Table S1). Second, the amounts of protein adsorbed observed with both systems (arrays and the individual resonators) are in good agreement with the literature. Focusing on the adsorption of Nav on gold (Figure 3b), we find 700 ± 180 ng/cm^2^ for the array and 590 ± 200 ng/cm^2^ for the individual sensors (Supplementary Table S1). The limiting value of the adsorbed Nav mass is also ~700 ng/cm^2^ (“limiting” here refers to the apparent saturation of the values of the adsorbed mass as a function of the adsorption step in Figure 3b,c). This is in good agreement with the literature values for Nav adsorption on gold that range between ~700 and ~1300 ng/cm^2^ (Supplementary Table S2). The variability here is due to the tendency of this protein to aggregate and corresponds to the age and treatment of the neutravidin solution. The aggregation tendency also explains the difference between the observed masses and their expected values based on the protein dimensions and between Nav and a very similar protein, streptavidin (Sav) (Supplementary Table S2) [36].

The amount of Nav adsorbed onto gold pre-coated with bBSA is significantly smaller, than directly onto gold (~400 ng/cm^2^, nearly identical for the arrays and the individual sensors; Figure 3c and Supplementary Table S1). Also, the variation in the mass of Nav adsorbed, expressed as a standard error, is 15 ng/cm^2^ when it is adsorbed on the bBSA layer, but 38 ng/cm^2^ when it is adsorbed onto gold directly, indicating that orienting Nav on a biotinylated substrate results in a more homogeneous layer. The value of the adsorbed mass of Nav on bBSA is in good agreement with that adsorbed on the biotinylated SLBs [36] (566 ng/cm^2^, Supplementary Table S2). One of the factors that contribute to the observed difference in the adsorbed mass is the difference in protein orientation: it is quasi-random when Nav is adsorbed on gold but fixed by the underlying bBSA layer when Nav adsorbs on bBSA. Such an effect has already been reported for Sav (Supplementary Table S2): a larger amount of this protein adsorbs on the mercaptounadecanoic acid (MUA) SAM or on gold than on a layer of biotinylated molecules (lipids or alkane thiols).

With Nav, there is the additional effect of aggregation of this protein, an effect that is absent in the case of Sav. Indeed, the difference between Nav adsorbed on gold and Nav adsorbed on bBSA or bSLB is much greater, than between streptavidin adsorbed on gold vs. bSLB or bSAM (Supplementary Table S2).

Notably, the tendency of neutravidin to aggregate appears to be reduced, when it is adsorbed in a fixed orientation on the biotinylated substrates (bBSA, Table S1, or bSLB, Supplementary Table S2), compared to when it is adsorbed on gold. This could also be due to the increased stability of this protein in the biotin-bound conformation [37], or to the steric limitations arising from the number of accessible biotins presented by the biotinylated surfaces. This effect is gradually lost in the subsequent adsorption steps (steps 2 and 3 in Supplementary Table S1 and Figure 4) since the limiting values of the adsorbed amounts of Nav are independent of the direction of the adsorption.

The value of the limiting mass of bBSA (~230 ng/cm^2^, steps 2 and 3 in Supplementary Table S1) nearly identical for the arrays and the individual sensors, is independent of the direction of adsorption, and is consistent with what has been reported by others for bBSA on Nav on gold or on Nav on SLBs (Table S2). It is worth noting that there is a significant difference in the behavior of the biotinylated and non-biotinylated versions of BSA on bare gold (c.f. our results quoted above with Table S2 in the Supporting Information). This has been noted before by Kim et al. when adsorbing BSA/bBSA mixtures on gold [38]. The effect has not been investigated further, but probably originates from the effect of biotinylation on the adsorbed protein orientation, conformation, or both.

We would like to underscore the importance of the comparison between the array and the individual sensors: since we [11,12,13,14,15,16] and others [17,39,40,41] have previously demonstrated the robustness and reproducibility of HFF resonators, the observed correspondence shows (1) that integration into an array does not affect their performance, and (2), demonstrates the functionality of the microfluidic cartridge. The comparison with the published results obtained with the same bioanalytical systems using classical, low-frequency individual sensors confirms that our array-based cartridge is capable quantitatively and accurately assaying bioanalytical systems based on specific binding.

#### 3.2.3. Biomolecular Transport in the Microfluidic Cartridge

**Figure 5:**
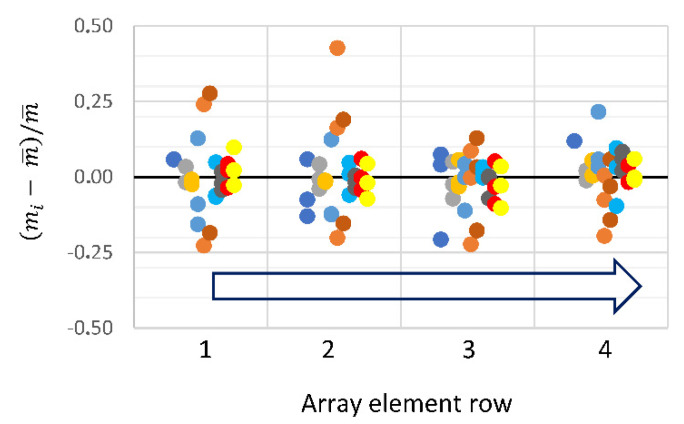
Variation in the adsorbed mass as a function of the sensor position. The absolute deviation of the adsorbed mass, normalized by the mean, is plotted against the row of the array element. $m_{i}$ is the adsorbed mass detected by the i-th element of the array (see Figure S1). $\bar{m}$ is the average mass in a given column. Different colors represent different experiments. Results of each individual experiment are offset from each other for visibility. Blue open arrow indicates the direction of the fluidic flow up the column.

**Figure 6:**
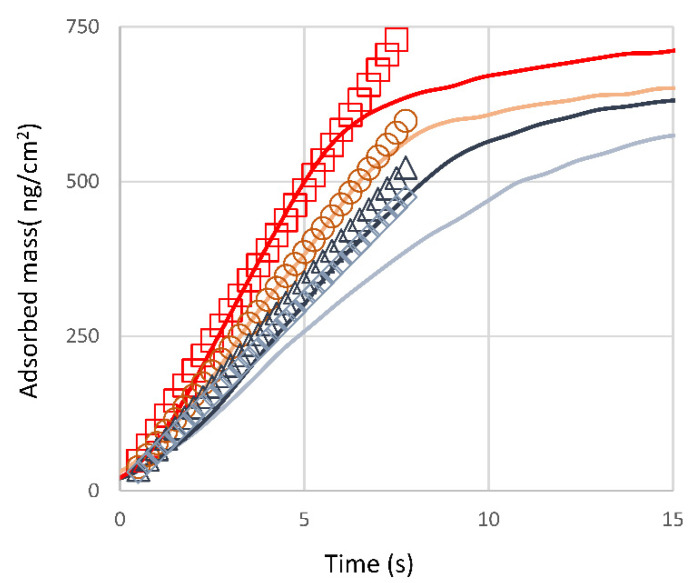
Comparison of the experimental and theoretical molecular transport rates at the array elements. Solid lines: experimental results for one array column obtained with Nav adsorbing on gold. Open symbols: calculated Γ(x,t), where x specifies the distance between the inlet and the array element.

One concern with an integrated array of sensing elements and the corresponding fluidics is the presence of systematic artifacts arising from transport limitations in the fluidic channels. Here, we first analyze the variation in the adsorbed protein layer masses detected by the different array elements (Figure 5) and then compare transport kinetics of the analytes in the fluidic cartridge with theoretical predictions based on molecular diffusion coefficients and the geometry of the fluidic channels and the arrays (Figure 6). We demonstrate lack of such systematic errors.

Indeed, it can be seen in Figure 5 that the distribution of the adsorbed masses of Nav as a function of the array element location is random. For most of the experiments, the variation is <15%, in terms of the absolute deviation. The data shown in Figure 5 also illustrate the robustness of the cartridge and array system to repeated cleaning and assembly cycles; arrays survive up to 30 such cycles without loss of performance.

Finally, we show that the experimentally observed transport rates can be compared semi-quantitatively with the predictions of simple calculations based on the channel geometry and molecular diffusion coefficient.

In the case of a flow through a rectangular channel with the width 2W and height 2R at a flow rate Q, the evolution of the surface coverage as a function of time and position along the channel, x, is described by Γ(x,t)=0.49 D23Q13R23W131x13Cb t, where Γ(x,t) is the adsorbed mass at location x and time t, D is the diffusion coefficient (60 µm^2^/s), and C*_b_* is the bulk analyte concentration [42,43]. This equation describes transport to a surface by a combination of flow and diffusion. The results are plotted in Figure 6 for our channel dimensions, Q = 4 µL/min, C*_b_* = 0.195 mg/mL and three different locations x, such that the first sensor is 1.8 mm from the inlet and the distance between the sensors is 1.8 mm. In the example shown in Figure 6, only the last of the four sensor elements displays significant deviation. The leveling off of the adsorption curves at longer times is due to surface saturation that is not taken into account by this equation, which only considers the transport of the molecules to the surface.

This equation applies to situations where surfaces act as “perfect sinks” (adsorption kinetics is transport-limited, or, equivalently, the rate, at which the molecule binds to the surface >> than the transport rate), and when the flow-mediated transport is much faster, than the diffusion-mediated transport so that the bulk concentration profile in the x-direction is uniform in time. The first of the two conditions is met in cases of quasi-irreversible adsorption of protein molecules at bare surfaces in the initial stages of the adsorption [44,45]. The second needs to be evaluated by comparing transport and diffusive timescales in our geometry. This can be formally done in terms of a pair of Peclet numbers, one comparing transport and diffusive timescales along the channel normal ($Pe_{H}=\frac{Q}{2DW}$.), and the other—along the channel length ($Pe_{S}=6\lambda^{2}Pe_{H}$, where $\lambda =L_{s}/2R$ is the ratio of sensor length to the channel height, and other variables are as defined above) [46]. For our geometry, for all relevant flow rates, both $Pe_{H}\gg 1$ and $Pe_{S}\gg 1$, indicating that indeed, the flow-mediated transport along the channel is much faster, than the diffusion across the channel, and substantiating the validity of the above equation for Γ(x,t). A more detailed analysis of the Peclet numbers for different analyte species can be found in Supplementary Section S3.

The differences between experiment and calculation, such as the one visible in Figure 6 for the last sensor in a column, must originate from the deviations of the channel geometry from that of a straight rectangular channel (inlet and outlet at 90° to the channel axis) and variation of the surface properties (advantageous contamination) between the sensor elements.

One further important assumption underpins the above analysis: that the material of the fluid cell is essentially non-adsorbing. This is because the well, where the analyte is placed (Figure 1, red circle), is located some 11 mm from the first sensor. However, it is impossible to fit the data for all four sensors if *x* for the first sensor is set to 11 mm, because the differences between sensor locations (1.8 mm apart) become irrelevant in comparison with the long inlet tube. The observed differences in the adsorption rates for the four sensors shown in Figure 6 therefore indicate that there is a minimal loss of the analyte on the inlet tube itself.

## 4. Conclusions

A microfluidic cartridge designed to host an array of 24 HFF-QCM resonators in bioanalytical applications was implemented. It works as a mechanical, electrical and thermal interface between the QCMD sensing array, the characterization instrument, and the fluid analyte. Currently, the assembly is aimed at research laboratories that reuse the array and allows fast and easy assembly and disassembly of the cartridge. Re-use of 30+ times with repeated surface cleaning is demonstrated. Use of low cost of the materials (PMMA, PDMS, custom PCB) makes the design appropriate for disposable applications. The functionality of the array + cartridge combination is evaluated using air-liquid shifts and adsorption of biomacromolecules (biotinylated albumin and neutravidin). Experimental results on the adsorbed masses and adsorption rates demonstrate quantitative agreement with the literature and theoretical considerations (protein geometry, transport conditions). Systematic errors arising from flow geometry are ruled out. The array + cartridge combination is used to show that the first adsorption step is critical in defining the molecular properties of the sensing interface: adsorbing neutravidin onto biotinylated BSA pre-adsorbed on the gold results in a more controlled layer of biotin sites for the subsequent sensing application than adsorbing neutravidin directly on the gold. In summary, the device is shown to be robust and function reliably in complex biosensing applications. In the future, it will be used to test human DNA samples for single base mutations in the colorectal cancer liquid biopsy samples and extended to immunoassays for pesticide and antibiotic detection in honey samples.

## Supplementary Materials

The following are available online at https://www.mdpi.com/2079-6374/10/12/189/s1, Element positions on the Monolithic 150 MHz HFF QCMD sensor array. Quantification of adsorbed protein masses with the array and the individual sensors. Literature values for the dimensions and adsorbed layer masses for BSA, bBSA, and Nav. Peclet numbers and depletion thickness layer calculation. Figure S1: Monolithic 150 MHz HFF-QCMD sensor arrays. Numbers indicate the i-th element of the array, where i = r +4(c − 1) with r = 1–4 and c = 1–6 row and column indexes, respectively. Table S1: Quantification of adsorbed protein masses. Table S2: Literature values for the dimensions and adsorbed layer masses for BSA, bBSA, and Nav. Table S3: Pe_H_ calculated for different flow rates and biomolecules. Table S4: 〖Pe〗_S calculated for different gasket deformation.
