# Supplementary material for: A Multichannel Microfluidic Sensing Cartridge for Bioanalytical Applications of Monolithic Quartz Crystal Microbalance
Source: Biosensors (Basel). 2020 Nov 24;10(12):189. doi: 10.3390/bios10120189 (PMC7760489; doi:10.3390/bios10120189)
Supplement: Supplementary file 1 [file biosensors-10-00189-s001.zip › SupportingInformation_Biosensors.docx]

Article

Multichannel Microfluidic Sensing Cartridge for Bioanalytical Applications. Supporting Information

María Calero, Román Fernández, Pablo García, José Vicente García, María García, Esther Gamero-Sandemetrio, Ilya Reviakine, Antonio Arnau and Yolanda Jiménez

S1. Monolithic 150 MHz HFF QCMD sensor arrays


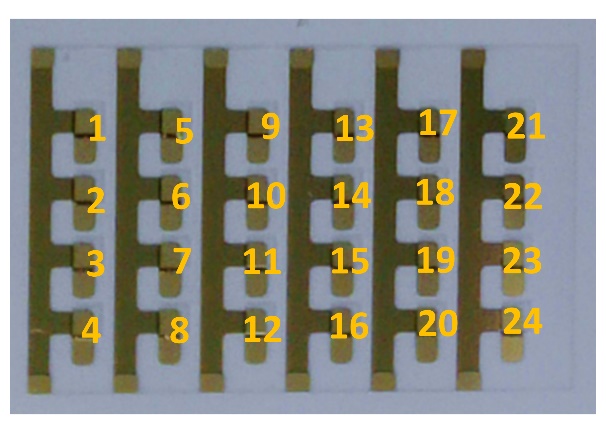


Figure S1. Monolithic 150 MHz HFF QCMD sensor arrays. Numbers indicate the i-th element of the array, where i = r +4(c – 1) with r = 1-4 and c = 1-6 row and column indexes, respectively.

S2. Protein Adsorption and Interaction Studies

**Table S1.** Quantification of adsorbed protein masses.

| **Step** | **Protein** | **Array** | **HFF** |  | **Protein** | **Array** | **HFF** |
| --- | --- | --- | --- | --- | --- | --- | --- |
|  |  | **Δm, ng/cm^2^** | **Δm, ng/cm^2^** |  |  | **Δm, ng/cm^2^** | **Δm, ng/cm^2^** |
| 1 | Nav | 700 ± 180 (23) | 590 ± 200 (12) |  | bBSA | 75 ± 73 (16) | 120 ± 100 (12) |
|  | BSA | 20 ± 30 (23) | 9 ± 5 (12) |  | BSA | 230 ± 58 (16) | 70 ± 42 (10) |
|  | bBSA | 340 ± 60 (15) | 250 ± 70(12) |  | Nav | 470  ± 60 (16) | 390 ± 180 (12) |
|  | BSA | 24 ± 5 (15) | 8 ± 5 (12) |  | BSA | − 11 ± 11 (8) | 6 ± 4 (12) |
| 2 | Nav | 780 ± 120 (23) | 700 ± 120 (12) |  | bBSA | 215 ± 34 (16) | 220 ± 60 (12) |
|  | BSA | 11 ± 10 (23) | 6 ± 4 (12) |  | BSA | 29 ± 17 (16) | 6 ± 4 (12) |
|  | bBSA | 250 ± 50 (23) | 220 ± 50 (12) |  | Nav | 670 ± 60 (16) | 670 ± 190 (11) |
|  | BSA | 3 ± 14(23) | 8 ± 5 (12) |  | BSA | 11 ± 7 (16) | 6 ± 5 (12) |
| 3 | Nav | 650 ± 30 (15) | 770 ± 140 (12) |  | bBSA | 250 ± 26 (16) | 220 ± 60 (12) |
|  | BSA | 8 ± 11(15) | 6 ± 4 (12) |  | BSA | 6 ± 9 (16) | 7 ± 5 (12) |
|  | bBSA | 190 ± 37 (15) | 280 ± 60 (8) |  | Nav | 700 ± 26 (8) | 660 ± 200 (11) |

Masses of the surface-adsorbed proteins calculated using the Sauerbrey[1] relationship from the frequency shifts for the arrays and the individual HFF resonators. On each type of sensor, the measurements were performed in two directions: starting from Nav (left columns) and starting from bBSA (right columns). Errors are standard deviations, number of individual measurements in the averages are shown in brackets. The data correspond to the plots shown in Figure 3 of the main manuscript.

**Table S2.** Literature values for the dimensions and adsorbed layer masses for BSA, bBSA, and Nav.

| **QCMD adsorbed layer masses [ng/cm^2^]** | | | | | **Dimensions [nm]^a^** | **Expected mass [ng/cm2]^b^** |
| --- | --- | --- | --- | --- | --- | --- |
| **Surface** | **Gold^c^** | **bSLB^c^** | **bSAM** | **MUASAM^c^** |  |  |
| Nav | 707 < ^c^; 908 – 1261^d^ | 566 < ^c^ | N/D | N/D | 6 × 5 × 4 | 460 – 650 |
| Sav | 530 ± 35 ^c^; 540 ^e^, 447 ^e^ | 495 ± 35 ^c^ | 446 ± 27 ^f^ | 575 ± 10 ^f^ |  |  |
| **Surface** | **Gold** | **Nav on gold** | **Nav on bSLBs** |  |  |  |
| BSA | 535 ± 92 ^g^ ;532h; ~ 450^i^ | N/A | N/A | ~ 450^i^ | 3 × 4 × 8 | 344 – 917 |
| bBSA | Table S1 | > 177 *^c^* | 168 ± 18*^c^* | N/A |  |  |

^a^ Sizes per molecule were directly obtained from the protein crystallographic dimensions, pdb codes 2avi and 4f5s for avidin and albumin, respectively. Both proteins are ~ 66 kDa. ^b^ Expected masses were calculated for the three different orientations assuming a homogeneous protein layer of a given height and a density ~ 1.15 g/cm^3^.[2] This approach takes into account that QCMD senses solvated, as opposed to dry, mass[3] and works well for streptavidin (Sav), where the corresponding dry masses that have been measured by SPR[4,5] and ellipsometry[6], are ~ 200 – 380 ng/cm^2^ for the oriented and random adsorption of streptavidin on biotinylated lipid layers or SAMs, respectively. ^c^ Wolny et al. [7] ^d^ Boujday et al.[8]; Tsortos et al.[9,10]; Hays et al. [11]; Kasper et al.[12]; ^e^ Höök et al. [2]; Larsson et al.[4]; ^f^ Su et al.[5] ^g^ Reimhult et al.[13] ^h^ Min et al.[14] ^i^ Thourson et al.[15]; N/A: not applicable. N/D: not determined.

S3. Peclet Numbers and Depletion Layer Thickness

Peclet numbers are a class of dimensionless numbers relevant in the study of mass transport phenomena. ${Pe}_{H}$refers to the Peclet number in the channel height direction and relates the rate of convection of a physical quantity by the flow to the rate of diffusion of the same quantity[16]:

| ${Pe}_{H}\equiv\frac{diffusive time}{convective time}\sim\frac{\left( 2R \right)^{2}/D}{\left( 2R \right)^{2}2W/Q}=\frac{Q}{D2W}$ | (S1) |
| --- | --- |

Where Q is the flow rate, D is the target molecule diffusivity, 2R is the channel height and 2W is the channel width. If ${Pe}_{H} \gg1$ convection dominates over diffusion, enhancing the mass transport and avoiding the propagation of the depletion layer. If ${Pe}_{H} \ll1$ , then diffusion dominates, and a depletion layer can propagate upwards in the flow. In our current design, width of the channel is mainly constrained by the spacing between HFF-QCM sensor columns in the array to 2W=1.46 mm. Maximum flow rate is limited by the fragility of the resonators to 100 µl/min. Commonly used samples in bioanalytic applications include proteins, antibodies, DNA or RNA. Diffusivity constant of these biomolecules varies widely in the range of 0.1 to 100 µm^2^s^-1^. Table S3 shows ${Pe}_{H}$calculated for different flow rates and analytes. As it can be observed, it is way larger than 1 for all the molecules and flow rates investigated.

**Table S3.** Pe_H_ calculated for different flow rates and biomolecules

|  | **Diffusivity**  **(µm^2^s^-1^)** | **PeH**  **(Q=15µl min^-1^)** | **PeH**  **(Q=50µl min^-1^)** | **PeH**  **(Q=100µl min^-1^)** |
| --- | --- | --- | --- | --- |
| Neutravidin[17] | 60 | 2854 | 9513 | 19026 |
| BSA,66 kDa, 4 nm[18] | 90 | 1903 | 6342 | 12684 |
| Liposomes 20 nm[18] | 25 | 6849 | 22831 | 45662 |
| mRNA (various sizes) max | 1 | 171233 | 570776 | 1141553 |
| DNA (2311 bp)[19] | 4,56 | 37551 | 125170 | 250340 |
| DNA (762 bp)[19] | 9,05 | 18921 | 63069 | 126138 |

A second Peclet number, ${Pe}_{S}$,can be used to estimate the thickness of the depletion layer, $\delta_{s}$[16]:

| $\delta_{s}\sim\frac{L_{s}}{{{Pe}_{S}}^{1/3}}$ | (S2) |
| --- | --- |

${Pe}_{S}$can be calculated according to next equation[16]:

| ${Pe}_{S}={6\lambda^{2}Pe}_{H}$ | (S3) |
| --- | --- |

Where, $\lambda$ is the ratio $\lambda={L_{s}}/{2R}$ where Ls is sensor length in the flow direction and 2Rthe channel height. Our HFF-QCM sensors have a lateral dimension of $L_{s}=550 \mu m$. Regarding the cartridge channel height, 2R, it is given by the thickness of the PDMS gasket (480 µm). Since PDMS is an elastic material, it is difficult to determine exactly its final thickness once the different parts of the cartridge are assembled and tightened. Table S4 shows ${Pe}_{S}$ and $\delta_{s}$values in two cases: (1) assuming no gasket deformation (2R=480 µm) and (2) assuming 20% gasket deformation (2R=380 µm).

**Table S4.** PeS calculated for different gasket deformation

| **2R (µm)** | **PeS** | **Depletion layer thickness (µm)** |
| --- | --- | --- |
| 480 | 29975,6 | 17,7 |
| 380 | 47828,2 | 15,1 |

References

1. Sauerbrey, G. Verwendung von Schwingquarzen zur Wägung dünner Schichten und zur Mikrowägung. *Zeitschrift für Phys.* **1959**, *155*, 206–222.

2. Höök, F.; Ray, A.; Nordén, B.; Kasemo, B. Characterization of PNA and DNA Immobilization and Subsequent Hybridization with DNA Using Acoustic-Shear-Wave Attenuation Measurements. *Langmuir* **2001**, *17*, 8305–8312.

3. Reviakine, I.; Johannsmann, D.; Richter, R.P. Hearing What You Cannot See and Visualizing What You Hear: Interpreting Quartz Crystal Microbalance Data from Solvated Interfaces. *Anal. Chem.* **2011**, *83*, 8838–8848.

4. Larsson, C.; Rodahl, M.; Höök, F. Characterization of DNA Immobilization and Subsequent Hybridization on a 2D Arrangement of Streptavidin on a Biotin-Modified Lipid Bilayer Supported on SiO 2. *Anal. Chem.* **2003**, *75*, 5080–5087.

5. Su, X.; Wu, Y.-J.; Robelek, R.; Knoll, W. Surface Plasmon Resonance Spectroscopy and Quartz Crystal Microbalance Study of Streptavidin Film Structure Effects on Biotinylated DNA Assembly and Target DNA Hybridization. *Langmuir* **2005**, *21*, 348–353.

6. Reiter, R.; Motschmann, H.; Knoll, W. Ellipsometric characterization of streptavidin binding to biotin-functionalized lipid monolayers at the water/air interface. *Langmuir* **1993**, *9*, 2430–2435.

7. Wolny, P.M.; Spatz, J.P.; Richter, R.P. On the Adsorption Behavior of Biotin-Binding Proteins on Gold and Silica. *Langmuir* **2010**, *26*, 1029–1034.

8. Boujday, S.; Bantegnie, A.; Briand, E.; Marnet, P.G.; Salmain, M.; Pradier, C.M. In-depth investigation of protein adsorption on gold surfaces: Correlating the structure and density to the efficiency of the sensing layer. *J. Phys. Chem. B* **2008**, *112*, 6708–6715.

9. Tsortos, A.; Papadakis, G.; Gizeli, E. Shear acoustic wave biosensor for detecting DNA intrinsic viscosity and conformation: A study with QCM-D. *Biosens. Bioelectron.* **2008**, *24*, 836–841.

10. Tsortos, A.; Papadakis, G.; Gizeli, E. Acoustic wave biosensor for detecting DNA conformation; A study with QCM-D. In Proceedings of the 2008 IEEE International Frequency Control Symposium; IEEE, 2008; Vol. 24, pp. 346–349.

11. Hays, H.C.W.; Millner, P.A.; Prodromidis, M.I. Development of capacitance based immunosensors on mixed self-assembled monolayers. *Sensors Actuators B Chem.* **2006**, *114*, 1064–1070.

12. Kasper, M.; Traxler, L.; Salopek, J.; Grabmayr, H.; Ebner, A. Broadband 120 MHz Impedance Quartz Crystal Microbalance ( QCM ) with Calibrated Resistance and Quantitative Dissipation for Biosensing Measurements at Higher Harmonic Frequencies. **2016**, 1–13.

13. Reimhult, K.; Petersson, K.; Krozer, A. QCM-D Analysis of the Performance of Blocking Agents on Gold and Polystyrene Surfaces. *Langmuir* **2008**, *24*, 8695–8700.

14. Min, H.; Freeman, E.; Zhang, W.; Ashraf, C.; Allara, D.; van Duin, A.C.T.; Tadigadapa, S. Modified Random Sequential Adsorption Model for Understanding Kinetics of Proteins Adsorption at a Liquid–Solid Interface. *Langmuir* **2017**, *33*, 7215–7224.

15. Thourson, S.B.; Marsh, C.A.; Doyle, B.J.; Timpe, S.J. Quartz crystal microbalance study of bovine serum albumin adsorption onto self-assembled monolayer-functionalized gold with subsequent ligand binding. *Colloids Surfaces B Biointerfaces* **2013**, *111*, 707–712.

16. Squires, T.M.; Messinger, R.J.; Manalis, S.R. Making it stick: convection, reaction and diffusion in surface-based biosensors. *Nat. Biotechnol.* **2008**, *26*, 417–426.

17. Wayment, J.R.; Harris, J.M. Biotin-avidin binding kinetics measured by single-molecule imaging. *Anal. Chem.* **2009**, *81*, 336–342.

18. Osidak, E.O.; Osidak, M.S.; Akhmanova, M.A.; Domogatskii, S.P. Collagen — A Biomaterial for Delivery of Growth Factors and Tissue Regeneration. *Russ. J. Gen. Chem.* **2014**, *84*, 102–113.

19. Pecora, R. Model Compound for Solution. *Science (80-. ).* **1991**, *251*, 893–898.

Publisher’s Note: MDPI stays neutral with regard to jurisdictional claims in published maps and institutional affiliations.

| 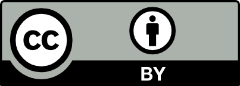 | © 2020 by the authors. Submitted for possible open access publication under the terms and conditions of the Creative Commons Attribution (CC BY) license (http://creativecommons.org/licenses/by/4.0/). |
| --- | --- |
