# Supplementary figures and images for: A Multichannel Microfluidic Sensing Cartridge for Bioanalytical Applications of Monolithic Quartz Crystal Microbalance
Source: Biosensors (Basel). 2020 Nov 24;10(12):189. doi: 10.3390/bios10120189 (PMC7760489; doi:10.3390/bios10120189)

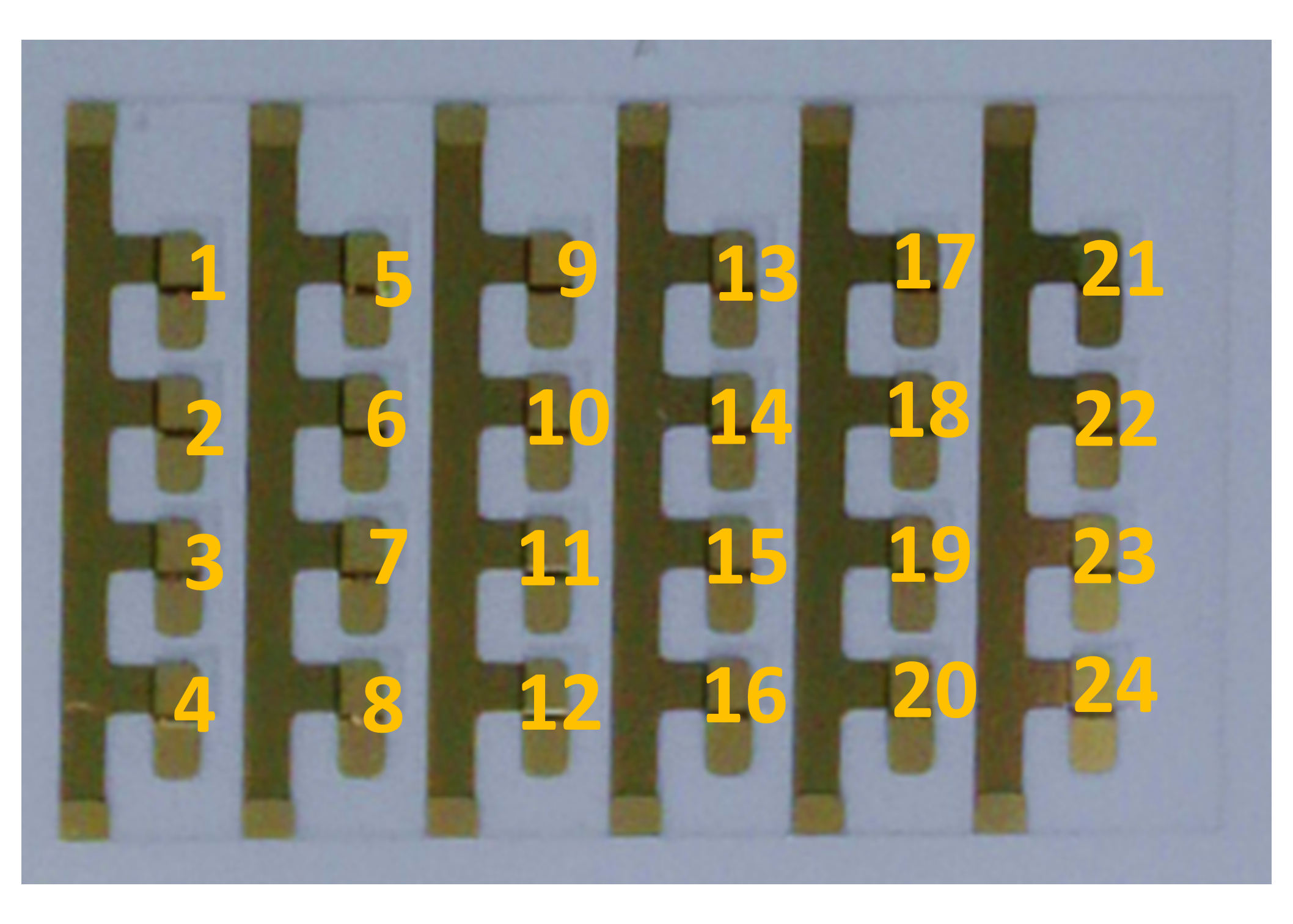

Supplement: Supplementary file 1 [file biosensors-10-00189-s001.zip › FigureS1.tif]
